# Supplementary material for: Effect of Traditional Chinese Exercises on Patients with Chronic Heart Failure (TCE-HF): A Systematic Review and Meta-Analysis
Source: J Clin Med. 2023 Mar 9;12(6):2150. doi: 10.3390/jcm12062150 (PMC10054845; doi:10.3390/jcm12062150)

SUPPLEMENTARY MATERIALS

1. Supplemental Methods

The search strategy was conducted on August 11, 2022.

Pubmed:

#1: ("heart failure"[Title/Abstract] OR "cardiac failure"[Title/Abstract] OR "heart decompensation"[Title/Abstract] OR "myocardial failure"[Title/Abstract] OR "congestive heart failure"[Title/Abstract]) AND (clinicaltrial[Filter] OR randomizedcontrolledtrial[Filter])

#2: ("tai chi"[Title/Abstract] OR "taiji"[Title/Abstract] OR "qigong"[Title/Abstract] OR "liuzijue"[Title/Abstract] OR "wuqinxi"[Title/Abstract] OR "yijinjing"[Title/Abstract] OR "baduanjin"[Title/Abstract] OR "traditional exercise"[Title/Abstract] OR "chinese traditional exercise"[Title/Abstract] OR ("raditional"[All Fields] AND "chinese exercise"[Title/Abstract]) OR "chinese exercise"[Title/Abstract] OR "kung fu"[Title/Abstract]) AND (clinicaltrial[Filter] OR randomizedcontrolledtrial[Filter])

#1 AND #2

Web of Science:

#1:((((TS=(heart failure)) OR TS=(Cardiac Failure)) OR TS=(Heart Decompensation)) OR TS=(Myocardial Failure)) OR TS=(Congestive Heart Failure) AND (DT==("CLINICAL TRIAL")

#2: ((((((((((TI=(tai chi)) OR TI=(taiji)) OR TI=(qigong)) OR TI=(liuzijue)) OR

TI=(wuqinxi)) OR TI=(yijinjing)) OR TI=(baduanjin)) OR TI=('traditional exercise')) OR TI=('Chinese traditional exercise')) OR TI=('Chinese exercise')) OR TI=(kung fu)) AND (DT==("CLINICAL TRIAL"))

#1 AND #2

Embase:

#1: ('heart failure':ab,ti OR 'cardiac failure':ab,ti OR 'heart decompensation':ab,ti OR 'myocardial failure':ab,ti OR 'congestive heart failure':ab,ti) AND ([controlled clinical trial]/lim OR [randomized controlled trial]/lim)

#2: ('tai chi':ab,ti OR taiji:ab,ti OR qigong:ab,ti OR liuzijue:ab,ti OR wuqinxi:ab,ti OR yijinjing:ab,ti OR baduanjin:ab,ti OR 'traditional exercise':ab,ti OR 'Chinese traditional exercise':ab,ti OR 'Chinese exercise':ab,ti OR ' kung fu ':ab,ti AND ([controlled clinical trial]/lim OR [randomized controlled trial]/lim)

#1 AND #2

Cochrane library:

#1: (tai chi):ti,ab,kw OR (taiji):ti,ab,kw OR (qigong):ti,ab,kw OR (liuzijue):ti,ab,kw OR (wuqinxi):ti,ab,kw (Word variations have been searched)

#2: (yijinjing):ti,ab,kw OR (baduanjin):ti,ab,kw OR (kung fu):ti,ab,kw OR ("chinese traditional exercise"):ti,ab,kw OR ("chinese exercise"):ti,ab,kw

#3: #1 OR #2

#4: ("heart failure"):ti,ab,kw OR ("cardiac failure"):ti,ab,kw OR ("heart decompensation"):ti,ab,kw OR ("myocardial failure"):ti,ab,kw OR ("congestive heart failure"):ti,ab,kw

#5: #3 AND #4

CNKI, Chongqing VIP, Wanfang Databases：

（心力衰竭 OR 心衰） AND （太极 OR 气功 OR 六字诀 OR 五禽戏 OR 八段锦 OR 易筋经 OR 功夫 OR 中国传统运动）

2. Supplementary Tables

Table S1. Characteristics of selected studies.

Author, Year	Region	Age, years. mean ± SD		NYHA class	Sample size (drop out)		HF etiology	Types of TCE (time/ frequency)	Control (time/ frequency)	Basic management	Duration, months	Outcome indicators
		TCE	CTL		TCE	CTL						
Wei D 2003	China	60.5±NA		II–IV	30	40	NA	Tai Chi (NA/7 times weekly)	-	TDs	3	LVEF
Yeh GY 2004	US	66±1 2	61±1 4	I–IV	15	15	Idiopathic dilated, ischemic, alcohol-related, hypertensive,	Tai Chi (60 min/twice weekly)	-	TDs; dietary, exercise advice	3	6MWD; BNP; MLHFQ; peak VO2; biomarkers

							peripartum, adriamycin-induced					
Barrow DM 2007	UK	68.4 ±NA	67.9± NA	II–III	32 (7)	33 (6)	NA	Tai Chi+Qigong (55 mins/twice weekly)	-	TDs; standard medical supervision	4	MLHFQ; Exercise capacity (ISWD); SCL-90
Yeh GY 2008	US	64.2 ±16.2	54.7± 11.8	I–III	8	10	NA	Tai Chi (60 min/twice weekly)	-	TDs; dietary, exercise advice	3	6MWD; MLHFQ
Yao CD 2010	China	52.4 ±6.32	51.7± 7.26	II	80	70	Idiopathic dilated, hypertensive, coronary atherosclerotic	Tai Chi (30 min/≥5 times weekly)	-	TDs; lifestyle guidance	6	6MWD; MLHFQ; LVEF; LVEDd

Caminiti G 2011	Italy	74.1 ±6	73.4± 2	II	30	30	Ischemic, idiopathic dilated	Tai Chi (30 min/twice weekly)	-	Cycling or walking (30 min/4 times weekly)	3	6MWD; grade of cardiac function; MacNewQLMI; NT-proBNP; Muscle Strength Measurement
Yeh GY 2011	US	68.1 ±11. 9	66.6± 12.1	I-III	50 (1)	50 (3)	Ischemic, nonischemic	Tai Chi (60 min/twice weekly)	Education module (60 min/twice weekly)	TDs; general exercise advice; education program	3	6MWD; BNP; MLHFQ; peak VO2; Exercise self-efficacy; Profile of Mood States; Biomarkers
Yeh GY 2013	US	68±1 1	63±1 1	I-III	8	8	NA	Tai Chi (60 min/twice weekly)	Aerobic exercise (60 min/twice weekly)	TDs; general exercise advice	3	6MWD; BNP; MLHFQ; peak VO2; LVEF; Exercise self-efficacy; Profile of Mood States
Li CF 2015	China	70.5±4.5		II–IV	90	90	NA	Yijingjin+Wuqinxi+Baduanjin (NA/NA)	-	TDs	12	6MWD; BNP; heart function classification
Sang L1 2015	China	65.3 ±8.2	76. 2±7. 5	II–III	50	50	Coronary atherosclerotic	Tai Chi (15 min/7 times weekly)	-	TDs	3	6MWD; MLHFQ; LVEF

Sang L2 2015	China	65.3±6.2	66.2±5.5	II–III	30	30	Coronary atherosclerotic	Tai Chi (15 min/7 times weekly)	-	TDs	3	BNP; LVEF; angiotensin II
Yang XJ 2015	China	70.50±4.85	68.53±4.93	II–IV	30	30	NA	NYHA I-II: Baduanjin (20-30 min/5-10 times weekly); NYHA III-IV: six tips (15-20min/ 5-10 times weekly)	-	Routine treatment and nursing	12	6MWD; BNP; grade of cardiac function; readmission rate; hospitalization costs per capita
Yu J 2015	China	72.14+8.37	71.52+8.43	II–IV	41	41	NA	NYHA I-II: Baduanjin (20-30 min/14 times weekly); NYHA III-IV: six tips (20-30 min/14 times weekly)	-	Routine treatment and nursing	12	6MWD; hospitalization costs per capita; grade of cardiac function; number of readmissions per capita
Zhou HM 2015	China	NA	NA	I–III	30 (0)	30 (1)	NA	Six tips (30 min/7 times weekly)	-	Health advice	6	6MWD
Pan XF 2016	China	67.7±12.11	66.2±11.8	II–III	31	30	NA	Tai Chi (30 min/7 times weekly)	-	TDs; health advice	6	6MWD; BNP; SF-36; LVEF; LVEDd; cardiac troponin T

Xiong XH 2016	China	70.3±6.4	69.7±7.2	II–III	33	30	Coronary atherosclerotic	Baduanjin (30 min/5 times weekly)	-	TDs	3	6MWD; NT-proBNP; MLHFQ; LVEF; readmission rate
Yan XF 2016	China	71.35±6.46	73.43±6.93	II–IV	36	36	NA	Six tips+Baduanjin (NA/NA)	-	Routine treatment and nursing	12	6MWD; BNP; grade of cardiac function
Yuan LH 2016	China	66.33±5.56	67.47±3.82	II–III	30 (2)	30 (1)	NA	Tai Chi (1-4 week: 15min/5 times weekly; 5-12week: 20-40min/ 5 times weekly)	-	Antidepressant treatment; mental advice	3	MLHFQ; HAMD; PSQI
Li RL 2017	China	69.24±6.56	66.56±5.31	II–III	30	30	NA	Baduanjin (NA/NA)	Walking training (NA/14 times weekly)	TDs; daily life care; dietary, mental advice	2	6MWD; MLHFQ

Zheng L 2017	China	59.45±7.20	59.10±9.10	II–III	11 (2)	8 (1)	NA	Six tips (30-40 min/7 times weekly)	-	TDs	3	6MWD; NT-proNP; MLHFQ; LVEF
Chen DM 2018	Taiwan	71.44±13.65	69.08±13.48	I–II	39 (9)	41 (8)	NA	Baduanjin (35 min/3 times weekly)	Usual care (NA/NA)	-	3	MLHFQ; Piper Fatigue Scale
Deng XJ 2018	China	64.7±4.2	67.2±4.9	I-III	57 (2)	56 (0)	Myocardial infarction	Tai Chi (40-60 min/5 times weekly)	-	TDs; daily exercise	6	6MWD; NT-proBNP; LVEF; HAMD
Hägglund L 2018	Sweden	75.6±NA	75.5±NA	II–III	25 (5)	20 (6)	NA	Tai Chi (60 min/twice weekly)	-	-	4	NT-proBNP; Multidimensional Fatigue Inventory; Short Physical Performance Battery
Lu HL 2019	China	69.0±6.8	68.6±7.6	III–IV	40	40	NA	Baduanjin (30 min/ 14 times weekly)	-	TDs; walking training (NA/14 times weekly)	12	MLHFQ

Pan W 2019	China	68.28±8.36	68.59±8.14	NA	42	42	NA	Baduanjin (NA/NA)	Walking training (NA/14 times weekly)	Daily life care; dietary, mental advice	2	6MWD; NT-proBNP; LVEF
Redwine LS. 2019	US	63±9	67±7	NA	25 (4)	23 (4)	NA	Tai Chi (Class Day: 60 min/twice weekly+ home training: 10- 20min per day)	-	Usual care	4	6MWD; BDI
Xu M 2019	China	55.49±10.24	56.34±11.59	III–IV	40	40	NA	Baduanjin (NA/NA)	Walking training (NA/14 times weekly)	-	4	6MWD; MLHFQ
Yu D 2019	China	65.51±7.04	65.77±7.12	II–III	49	49	NA	Tai Chi (30 min/7 times weekly) + Baduanjin (30 min/4-5 times weekly)	-	TDs; dietary advice	3	6MWD; peak VO2; LVEF; LVEDd; LVESd; E/A; SF-36

Yu T 2019a	China	58.8±11.2	61.8±12.7	I-III	40	40	Ischemic, nonischemic	Tai Chi (30 min/≥5 times weekly)	Walking training (30 min/≥5 times weekly)	TDs; dietary, exercise advice	5.6	6MWD; BNP; LVEF; LVEDd
Yu T 2019b	China	59.4±12.1	61.8±12.7	I-III	40	40	Ischemic, nonischemic	Tai Chi (60 min/≥5 times weekly)	Walking training (30 min/≥5 times weekly)	TDs; dietary, exercise advice	5.6	6MWD; BNP; LVEF; LVEDd
Zhou H 2019	China	65.78±5.32	66.54±4.43	II–IV	34	34	NA	Baduanjin (30min/14 times weekly)	-	TDs; daily life care; dietary, mental advice	NA	6MWD; ADL
Jiao YL 2020	China	66.6±6.32	64.32±5.23	II–III	40	40	NA	Baduanjin (10min/14 times weekly)	-	TDs	6	6MWD; MLHFQ; LVEF; NT-proBNP

Ke JH 2020	China	65.33±NA	67.80±NA	II–III	30	30	NA	Baduanjin (60min/5 times weekly)	-	TDs	6	LVEF; LVESV; LVEDV; biomarkers
Yao LY 2020	China	56.80±6.1	56.20±6.3	NA	25	25	NA	Baduanjin (NA/NA)	-	TDS; dietary, mental advice	3	MLHFQ; LVEF; LVEDd; LVESd
Yu ML 2020	China	67.7±5.8	68.3±6.4	II–III	100 (3)	100 (6)	Myocardial infarction	Tai Chi (20 min/14 times weekly)	-	TDs; dietary, mental advice	6	6MWD; BNP; MLHFQ; LVEF; aldosterone; readmission rate; mortality rate; grade of cardiac function
Zhou B 2020	China	62.17±5.28	62.03±6.31	II–III	52	51	NA	Tai Chi (20-40 min/5 times weekly)	-	TDs; health, dietary advice	3	MLHFQ; LVEF; LVEDd; LVESd; HAMD; HAMA; SDS; PSQI

Deng LM 2021	China	65.42 ± 3.71	65.54 ± 3.68	II–III	60	50	NA	Baduanjin (NA/7 times weekly)	Walking training (20-30min/7 times weekly)	TDs; health advice; lifestyle guidance	12	BNP; LVEDd; LVESd
Kang ZL 2021	China	68.36±5.03	69.25±5.33	II–III	38	38	NA	Baduanjin (30min/7 times weekly)	-	TDs; walking training (1-4 week: 30min/5 times weekly; 5-24week: 45min/ 5 times weekly)	6	6MWD; NT-proNP; MLHFQ; HDMD; HDMA; LVEF
Yang HX 2021	China	69.98±5.49	71.32±6.24	I-III	54 (7)	54 (7)	NA	Tai Chi (60 min/3-6 times weekly)	-	TDs	12	6MWD; Time up and go test; NT-proBNP; LVEF; LVEDd; E/A
Ye L 2021	China	63.22±18.72	65.12±19.91	II–III	40	40	NA	Baduanjin (30min/3-5 times weekly)	-	TDs; health advice; exercise	6	MLHFQ; peak VO2

Zhou YX 2021	China	57.87±4.61	58.41±4.52	II–III	50	50	Myocardial infarction	Baduanjin (30min/14 times weekly)	-	TDs; dietary, mental advice	3	6MWD; MLHFQ; LVEF; LVEDd; LVESd

TCE: traditional Chinese exercises; CTL: control; SD: standard deviation; NYHA: New York Heart Association; LVEF: left ventricular ejection fraction; HF: heart failure TD: therapeutic drugs (prescribed according to heart failure management guideline); 6MWD: 6-minute walking distance; BNP: B-type natriuretic peptide; NT-proBNP: N-terminal pro-B-type natriuretic peptide; MLHFQ: Minnesota Living with Heart Failure Questionnaire; peak VO2: peak oxygen consumption; ISWD: incremental shuttle walk test; LVEDd: left ventricular end-diastolic dimension; BDI: Beck depression inventory; LVESd: left ventricular end-systolic diameter; E/A: the ratio value of peak systolic velocity in early diastolic phase by peak systolic velocity in late diastolic phase; SF-36: 36-Item Short Form; ADL: daily life ability scale; LVESV: left ventricular end-systolic volume; LVEDV: left ventricular end-diastolic volume; HAMD: Hamilton depression scale; HAMA: Hamilton anxiety scale; SDS: self-depression rating scale; PSQI: Pittsburgh after treatment Sleep Quality Index; NA: not available.

Table S2. The baseline and final values of the TCE group in selected studies.

Author, Year	6MWD (m±SD)		LVEF (%±SD)		MLHFQ (±SD)		Peak VO2 (mL/kg/min±SD)		NTpro-BNP (pg/mL±SD)		BNP (pg/mL±SD)	
	baseline	final	baseline	final	baseline	final	baseline	final	baseline	final	baseline	final
Wei D 2003	NA	NA	45.42±2.62	53.90±4.68	NA	NA	NA	NA	NA	NA	NA	NA
Yeh GY 2004	327±106	412±116	24±7	NA	43±21	26±23	10.5±3	11.4±3	NA	NA	329±377	281±385
Barrow DM 2007	NA	NA	NA	NA	33	18.1	NA	NA	NA	NA	0.235±NA	NA
Yeh GY 2008	change: 76±52		25 ± 6	NA	change: -17±14		NA	NA	NA	NA	NA	NA

Yao CD 2010	374±81	554±94	30.85±9.78	48.63±9.37	51.6±18.4	32.6±14.5	NA	NA	NA	NA	NA	NA
Caminiti G 2011	214.9±32	291.5±46	33.6±9	NA	NA	NA	NA	NA	136.4 ± 31	99.7 ± 22	NA	NA
Yeh GY 2011	391±NA	426±NA	28.3±8.0	NA	NA	NA	11.9±NA	13.0±NA	NA	NA	102±NA	92±NA
Yeh GY 2013	335.4±174	404.2±190	62±9	62±9	32.8±18	28.7±16	14.5±7	15.2±6	NA	NA	98±85	98±116
Li CF 2015	302.6±84.9	409.6±89.2	NA	NA	NA	NA	NA	NA	NA	NA	345.6±93.6	215.3±79.9
Sang L1 2015	312. 2±130. 9	439. 7±80. 2	36. 4±3. 0	56. 2±3. 8	43. 8±1. 6	32. 7±1. 9	NA	NA	NA	NA	NA	NA

Sang L2 2015	NA	NA	35.4±3.1	55.4±3.3	NA	NA	NA	NA	NA	NA	67.3±9.2	31. 8 ± 3.8
Yang XJ 2015	322.67±124.97	409.67±139.22	NA	NA	NA	NA	NA	NA	NA	NA	345.60±293.63	245.33±249.91
Yu J 2015	NA	412.56±138.26	NA	NA	NA	NA	NA	NA	NA	NA	NA	NA
Zhou HM 2015	209.14±133.95	341.38±121.79	≤40%	NA	NA	NA	NA	NA	NA	NA	NA	NA
Pan XF 2016	371.0±26.0	415.0±38.0	32.4±7.2	36.3±9.3	NA	NA	NA	NA	NA	NA	432.0±57.0	223.0±29.0
Xiong XH 2016	316.8±42.3	512.5±50.2	40.2±5.3	48.9±5.8	35.8±1.7	11.2±1.8	NA	NA	4632.3±892.8	1329.4±567.3	NA	NA

Yan XF 2016	NA	413.58±153.54	NA	NA	NA	NA	NA	NA	NA	NA	NA	249.51±229.34
Yuan LH 2016	NA	NA	NA	NA	46.00±1.91	37.71±2.37	NA	NA	NA	NA	NA	NA
Li RL 2017	189.21±21.42	550.60±40.51	NA	NA	68.62±10.25	41.72±11.46	NA	NA	NA	NA	NA	NA
Zheng L 2017	370.00±77.04	445.77±46.67	51.80±4.36	58.78±12.81	38.00±8.42	17.33±1.87	NA	NA	1618.27±2176.07	715±964.23	NA	NA
Chen DM 2018	NA	NA	60.44±13.35	NA	22.97±17.86	15.40±11.74	NA	NA	NA	NA	NA	NA
Deng XJ 2018	261.2 ±52.9	558.5 ±67.1	42.7 ±6.1	49.0 ±5.4	NA	NA	NA	NA	3904.0±772.4	851.0 ±180.4	NA	NA

Hägglund L 2018	393±NA	NA	NA	NA	39.4±22.6	NA	NA	NA	4479±7309	3279 ± 3448	NA	NA
Lu HL 2019	NA	NA	NA	NA	28.40±2.42	9.96±2.18	NA	NA	NA	NA	NA	NA
Pan W 2019	126. 16 27.95	368.45±59. 71	40.26±5.38	48.97±5. 61	NA	NA	NA	NA	4632. 38 ± 859. 47	1328. 46 ± 534. 79	NA	NA
Redwine LS 2019	285.90 ± 109.42	236.52±NA	44± 13	NA	NA	NA	NA	NA	NA	NA	NA	NA
Xu M 2019	163.85±27.49	576.43±64.92	NA	NA	78.56±6.93	42.31±3.48	NA	NA	NA	NA	NA	NA
Yu D 2019	291.95±31.09	398.57±41.90	42.33±4.69	51.91±5.52	NA	NA	721.03±75.44	901.56±92.83	NA	NA	NA	NA

Yu T 2019 a	313.3±86.3	384.9±84.2	31.5±8.6	40.4±7.9	NA	NA	NA	NA	NA	NA	325.5±133.4	161.5±80.2
Yu T 2019 b	316.6±89.0	398.4±88.7	30.8±8.8	42.4±9.2	NA	NA	NA	NA	NA	NA	334.5±137.0	138.7±63.2
Zhou H 2019	189.69±36.78	568.58±46.47	NA	NA	NA	NA	NA	NA	NA	NA	NA	NA
Jiao YL 2020	216.79±50.05	330.65±40.42	40.21±7.29	57.25±8.45	5.69±7.16	15.25±10.04	NA	NA	1143.47±264.47	791.74±145.27	NA	NA
Ke JH 2020	NA	NA	46.8±4.2	51.6±2.7	NA	NA	NA	NA	NA	NA	NA	NA
Yao LY 2020	NA	NA	NA	50.34±12.88	NA	NA	NA	NA	NA	NA	NA	NA
Yu ML 2020	182.7±39.3	350.9±41.0	46.4±3.2	54.2±6.7	80.5±9.5	54.8±10.3	NA	NA	NA	NA	462.7±35.8	259.3±10.6

Zhou B 2020	NA	NA	41.19±4.63	52.06±5.10	45.60±3.14	36.67±2.11	NA	NA	NA	NA	NA	NA
Deng LM 2021	NA	NA	NA	NA	NA	NA	NA	NA	NA	NA	896.33±21.99	85.48±12.47
Kang ZL 2021	258.98±18.33	336.36±36.80	39.93±3.98	50.33±6.39	76.03±12.28	55.03±9.09	NA	NA	698.83±83.98	409.89±80.99	NA	NA
Yang HX 2021	422.09±64.55	465.79±91.33	51.81±3.15	52.43±2.58	NA	NA	NA	NA	917.47±202.62	876.02±81.10	NA	NA
Ye L 2021	NA	NA	NA	NA	42.18±8.75	31.39±7.21	16.15±1.81	18.89±1.63	NA	NA	NA	NA
Zhou YX 2021	360.85±21.64	498.52±47.58	41.25±11.41	51.24±12.04	NA	NA	NA	NA	NA	NA	NA	NA

NA: not available.

Table S3. The baseline and final values of the control group in selected studies.

Author, Year	6MWD (m±SD)		LVEF (%±SD)		MLHFQ (±SD)		Peak VO2 (mL/kg/min±SD)		NTpro-BNP (pg/mL±SD)		BNP (pg/mL±SD)	
	baseline	final	baseline	final	baseline	final	baseline	final	baseline	final	baseline	final
Wei D 2003	NA	NA	46.28±3.08	45.20±2.38	NA	NA	NA	NA	NA	NA	NA	NA
Yeh GY 2004	340±117	289±165	22±8	NA	44±20	52±25	11.1±6	10.4±6	NA	NA	285±340	375±429
Barrow DM 2007	NA	NA	NA	NA	34	31.6	NA	NA	NA	NA	0.239±NA	NA
Yeh GY 2008	change: -33±85		23 ± 9	NA	change: 7±10		NA	NA	NA	NA	NA	NA

Yao CD 2010	371±87	461±102	30.22±9. 32	39.62±7. 28	52.2±17. 3	45.4±12. 2	NA	NA	NA	NA	NA	NA
Caminiti G 2011	219.2±23	272.0±33	32.8±12	NA	NA	NA	NA	NA	134.5 ± 28	111.7 ± 24	NA	NA
Yeh GY 2011	392±NA	394±NA	29.8±7.3	NA	NA	NA	13.5±NA	13.0±NA	NA	NA	106±NA	119±NA
Yeh GY 2013	349.7±216	360.1±205	65±8	64±7	42.0±30	28.6±25	13.1±5	13.0±4	NA	NA	72±85	107±99
Li CF 2015	301.0±88. 8	334.8±84. 5	NA	NA	NA	NA	NA	NA	NA	NA	336.0±93. 2	272.4±87. 9
Sang L1 2015	343. 8±135	405. 6±110. 6	37. 0±2. 8	41. 8±3. 7	43. 2±1. 5	40. 3±1. 4	NA	NA	NA	NA	NA	NA

Sang L2 2015	NA	NA	36. 5 ± 2. 6	42.8±3.3	NA	NA	NA	NA	NA	NA	66.8±9.1	42.5±5.9
Yang XJ 2015	341.00±128.18	314.83±124.52	NA	NA	NA	NA	NA	NA	NA	NA	336.00±303.26	372.40±277.94
Yu J 2015	NA	315.26±123.52	NA	NA	NA	NA	NA	NA	NA	NA	NA	NA
Zhou HM 2015	205.86±124.82	252.72±124.18	≤40%	NA	NA	NA	NA	NA	NA	NA	NA	NA
Pan XF 2016	370.0±24.0	369.0±22.0	33.1±7.4	33.9±7.7	NA	NA	NA	NA	NA	NA	431.0±56.0	404.0±49.0
Xiong XH 2016	312.1±45.6	436.2±49.8	41.5±5.8	44.7±4.9	35.2±2.1	19.7±1.9	NA	NA	4556±913.3	1373.2±552.1	NA	NA

Yan XF 2016	NA	322.12±128.36	NA	NA	NA	NA	NA	NA	NA	NA	NA	382.16±279.52
Yuan LH 2016	NA	NA	NA	NA	46.28±1.53	43.04±2.15	NA	NA	NA	NA	NA	NA
Li RL 2017	191.30±32.17	368.71±32.24	NA	NA	67.87±11.44	59.38±12.61	NA	NA	NA	NA	NA	NA
Zheng L 2017	304.50±89.67	352.8±85.02	48.60±7.54	64.24±17.64	45.00±12.23	38.00±11.57	NA	NA	1307.62±1693.28	955.7±1187.5	NA	NA
Chen DM 2018	NA	NA	56.68±17.56	NA	19.21±13.47	20.33±12.35	NA	NA	NA	NA	NA	NA
Deng XJ 2018	275.1 ±47.2	430.8 ±57.6	41.0 ±5.4	46.0 ±5.2	NA	NA	NA	NA	3651.0 ±557.9	1583.8 ±221.9	NA	NA

Hägglund L<br>2018	358±NA	NA	NA	NA	45.2±24.<br>3	NA	NA	NA	3379±7332	2736 ± 2594	NA	NA
Lu HL 2019	NA	NA	NA	NA	28.56±2.<br>12	11.16±2.<br>26	NA	NA	NA	NA	NA	NA
Pan W 2019	130.<br>69±30. 52	327.<br>92±62. 33	41.58±5.<br>43	43.<br>97±5. 78	NA	NA	NA	NA	4687. 42 ±<br>893. 34	1685. 47 ±<br>549. 21	NA	NA
Redwine LS<br>2019	308.15±02<br>.41	245.66±N<br>A	46± 12	NA	NA	NA	NA	NA	NA	NA	NA	NA
Xu M 2019	170.46±30<br>.55	467.81±57<br>.22	NA	NA	75.64±7.<br>02	51.46±5.<br>17	NA	NA	NA	NA	NA	NA
Yu D 2019	292.44±30<br>.78	349.13±36<br>.33	42.41±4.<br>71	46.77±5.<br>03	NA	NA	721.22±7<br>5.17	812.34±8<br>2.05	NA	NA	NA	NA

Yu T 2019 a	309.7±84.7	365.3±75.7	32.2±8.4	38.8±8.2	NA	NA	NA	NA	NA	NA	314.5±121.7	191.2±80.6
Yu T 2019 b	309.7±84.7	365.3±75.7	32.2±8.4	38.8±8.2	NA	NA	NA	NA	NA	NA	314.5±121.7	191.2±80.6
Zhou H 2019	193.34±32.53	367.47±48.59	NA	NA	NA	NA	NA	NA	NA	NA	NA	NA
Jiao YL 2020	220.58±47.98	291.62±31.68	40.25±8.26	49.23±7.12	35.72±7.13	25.21±4.69	NA	NA	1138.31±278.54	793.74±144.68	NA	NA
Ke JH 2020	NA	NA	48.5±4.0	49.6±1.6	NA	NA	NA	NA	NA	NA	NA	NA
Yao LY 2020	NA	NA	NA	42.40±12.66	NA	NA	NA	NA	NA	NA	NA	NA
Yu ML 2020	184.2±36.6	286.7±34.9	46.5±4.5	50.6±5.4	80.6±10.4	68.5±12.5	NA	NA	NA	NA	460.3±36.8	312.7±34.5

Zhou B 2020	NA	NA	41.07±5.38	48.72±4.88	45.78±3.59	41.08±2.19	NA	NA	NA	NA	NA	NA
Deng LM 2021	NA	NA	NA	NA	NA	NA	NA	NA	NA	NA	895.48±22.57	98.75±11.65
Kang ZL 2021	263.36±16.36	316.30±30.98	40.33±3.66	46.89±5.93	72.98±11.59	62.80±8.86	NA	NA	682.69±69.98	463.80±93.88	NA	NA
Yang HX 2021	400.53±69.18	420.47±79.09	50.95±2.69	51.99±2.52	NA	NA	NA	NA	923.11±223.07	932.23±155.85	NA	NA
Ye L 2021	NA	NA	NA	NA	43.45±8.92	40.98±8.77	16.23±1.72	16.31±1.95	NA	NA	NA	NA
Zhou YX 2021	354.52±20.76	421.65±40.65	43.86±14.84	45.71±14.25	NA	NA	NA	NA	NA	NA	NA	NA

NA: not available.

Table S4. The characteristics of each type of TCE.

	Characteristics	Benefits
Tai Chi	Tai Chi is a type of low-intensity aerobic exercise characterized by gentle movements designed to dissipate force throughout the body while the subject changes pose, Tai Chi features gentle, coordinated and smooth movements of the body, stressing constantly shifting of body weight between two legs with both knees slightly flexed. Tai chi has been estimated to equal about 2-4 metabolic equivalents, comparable with mild-moderate aerobic exercise.	Tai chi can strengthen the joints, modulate pressure receptors in the aortic arch and carotid sinus. It has shown benefits in improving health-related fitness, balance, mental control, cardiorespiratory function, muscle strength of lower extremities.

Qigong	Qigong is an umbrella term covering a spectrum of mind-body exercises, such as Dao-Yin-Shu (physical and breathing exercise), Wuqinxi, Baduanjin, and Yijining.	By using slow and gentle physical movements and synchronizing breath with meditation, it can coordinate breathing, stretch body, relax muscles, and regulate attention and consciousness.
Baduanjin	Baduanjin, a type of aerobic Qigong exercises with simple, slow and relaxing movements, consists of eight set of actions including support heaven with both hands, dragon sprays water with force, big bird spreads its wings, lift window to look at the moon on the left, descend to earth with force, beautiful maiden twists her waist to the right, extend shoulders to bring hands together and dragon claws to	Baduanjin can reduce oxygen consumption of myocardial by regulating the vital energy of collateral channels and organs in the body, relieving the cardiac load, and improving the body’s ability to transport and utilize oxygen in blood circulation. It can inhibit the formation of free radicals, improve vascular elasticity, and reduce blood viscosity to ensure the normal flow of blood.

	the left.	
Yijinjing	Yijinjing focus on the center of the body mass, shift of garment, moderate knee flexion, extension and rotation movement, and coupled with breathing exercises, generally at the movement of knee flexion with inhalation. It emphasizes the combination of symmetrical physical postures, meditative mind, and breathing techniques in a harmonious manner.	With simplified patterns and directions, Yijinjing is easy to practice with few limitations.Yijinjing can improve physiological function, and movement disorders mental health as well as enhance organs function and immunity ability.
Wuqinxi	Wuqinxi imitates the movement and breathing patterns of five animals (tigers, deer, bears, monkeys, and birds) and emphasizes the integration of body, breath,	Through muscle activity around the hip and ankles, Wuqinxi can dredge systemic blood circulation.

	and mind.	
Liuzijue	Liuzijue consists of a combination of respiratory patterns that involves abdominal breathing and pursed lip breathing. Six sounds (Xu, He, Hue, Si, Tap and Xi) are produced with different body movements.	Liuzijue's type of breathing slows the expiratory flow rate, which especially facilitates gas exchange in patients with chronic obstructive pulmonary diseases.

TCE: traditional Chinese exercise

3. Supplementary Figures

Figure S1. Risk of bias assessment of included studies.

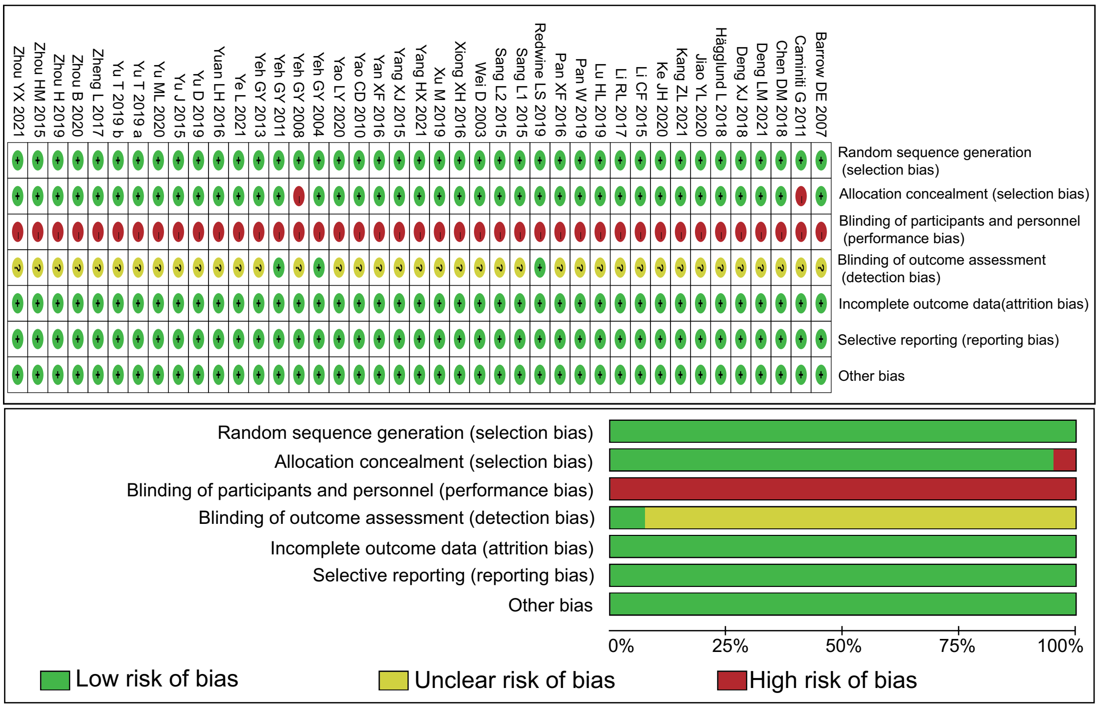

Figure S2. Publication bias of all the outcomes. (**A**) 6-minute walk distance (6MWD), (**B**) B-type natriuretic peptide (BNP), (**C**) left ventricular ejection fraction (LVEF); (**D**) Minnesota Living with Heart Failure Questionnaire (MLHFQ), (**E**) N-terminal pro-B natriuretic peptide (NT-proBNP), (**F**) peak oxygen consumption (peak VO2).

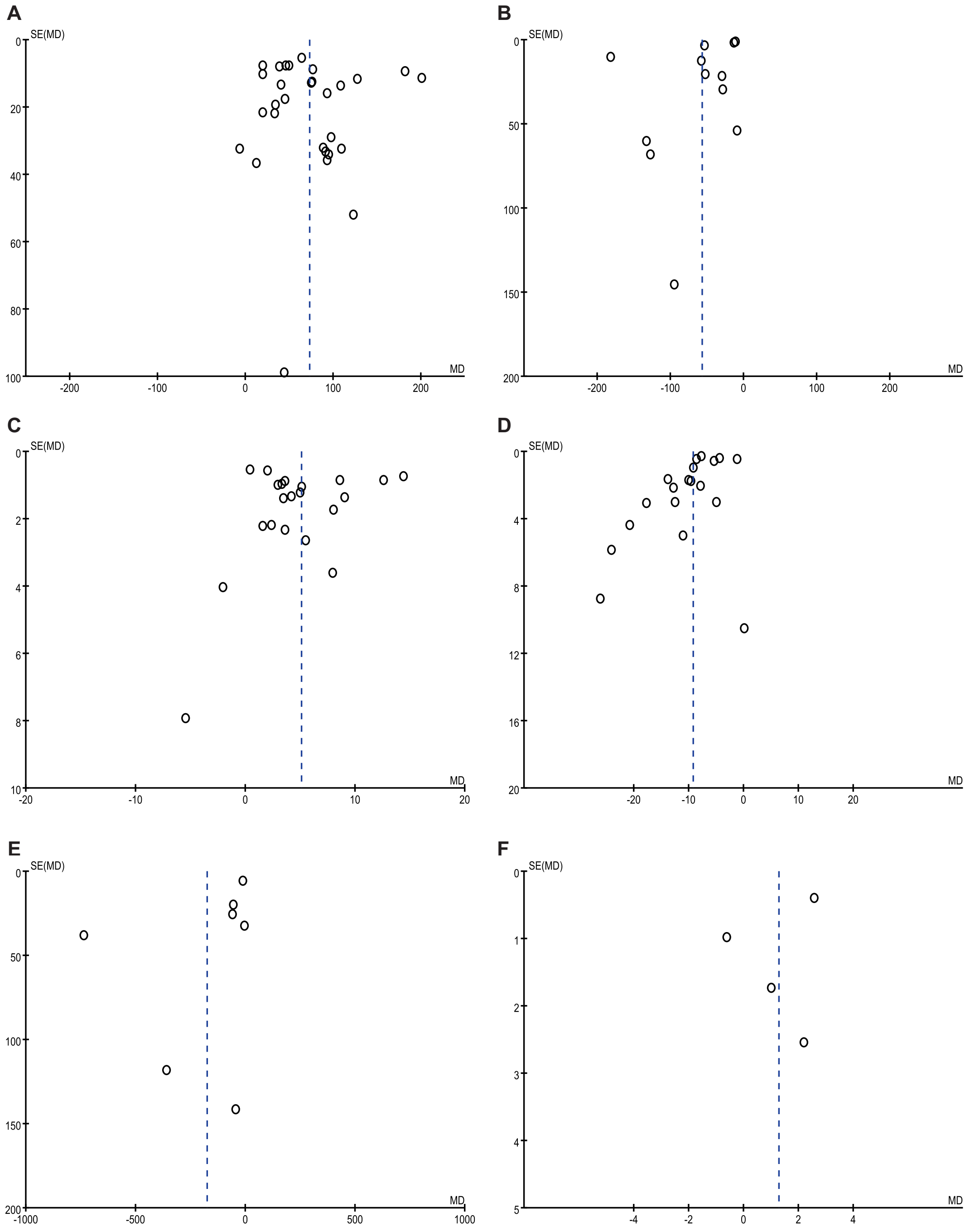

Figure S3. Sensitivity of all the outcomes. (**A**) 6-minute walk distance (6MWD), (**B**) B-type natriuretic peptide (BNP), (**C**) left ventricular ejection fraction (LVEF);(**D**) Minnesota Living with Heart Failure Questionnaire (MLHFQ), (**E**) N-terminal pro-B natriuretic peptide (NT-proBNP), (**F**) peak oxygen consumption (peak VO2).

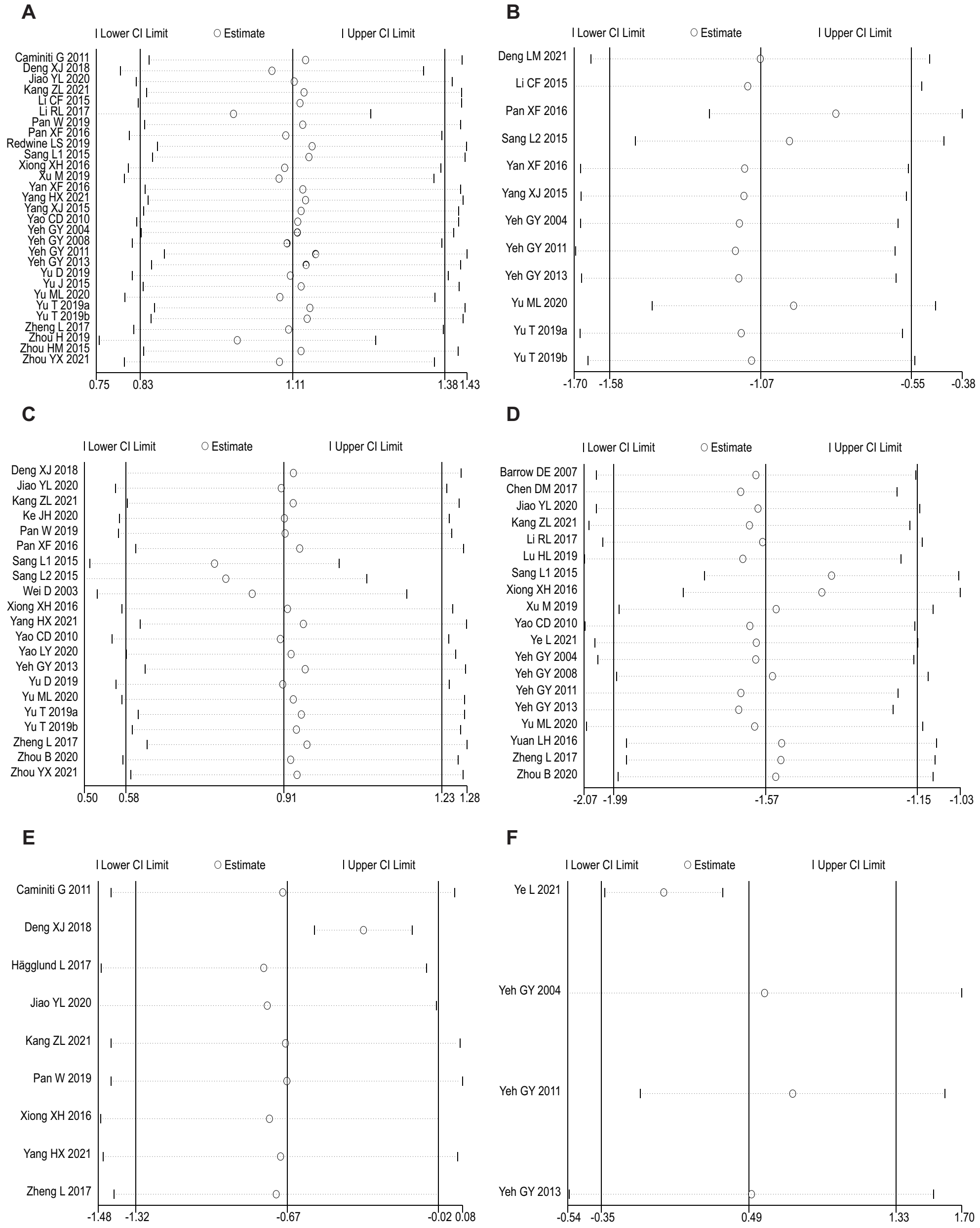

Supplement: Supplementary file 1 [file jcm-12-02150-s001.zip › jcm-2213398-supplementary.pdf]
